# Supplementary material for: Reconciling Mining with the Conservation of Cave Biodiversity: A Quantitative Baseline to Help Establish Conservation Priorities
Source: PLoS One. 2016 Dec 20;11(12):e0168348. doi: 10.1371/journal.pone.0168348 (PMC5173368; doi:10.1371/journal.pone.0168348)
Supplement: S1 Dataset — (ZIP) [file pone.0168348.s002.zip › Taxa/Serra Norte/SN_2013/N4WS/Lista N4WS-18.pdf]

CAVIDADE N4WS-0018/0048

Classe	Ordem	Fam/Outros	Gên/Outros	Espécie	S-U	U-S	Zon.
Arachnida	Acari	(Oribatida)		sp.1		X	P
Arachnida	Acari	Argasidae	*Ornithodoros*	sp.1		X	P
Arachnida	Amblypygi	Phrynidae	*Heterophrynus*	*longicornis*	2	1	P
Arachnida	Araneae	Araneidae	*Alpaida*	*septemmammata*	X		E
Arachnida	Araneae	Caponiidae		jovem		X	P
Arachnida	Araneae	Ochyroceratidae		jovem	X	X	P
Arachnida	Araneae	Pholcidae		jovem	X		E
Arachnida	Araneae	Salticidae		jovem	X		E
Arachnida	Araneae	Scytodidae	*Scytodes*	*globula*	2	4	E/P
Arachnida	Araneae	Theridiosomatidae	*Plato*	sp.1	X	X	P
Arachnida	Opiliones	Sclerosomatidae	*Prionostema*	sp.1	X		E
Arachnida	Pseudoscorpiones	Chernetidae	*Spelaeochernes*	sp.1	X	X	E/P
Chilopoda	Scolopendromorpha	Scolopocryptopidae	*Newportia*	sp.1	1		E
Chilopoda	Scolopendromorpha	Scolopocryptopidae	*Tidops*	sp.1	1		E
Chilopoda	Scutigeromorpha	Pselliodidae		jovem	1		E
Diplopoda	Polydesmida	Chelodesmidae		sp.1	1		P
Diplopoda	Polydesmida	Pyrgodesmidae		sp.1	X		P
Diplopoda	Polyxenida	Hypogexenidae		sp.1		X	P
Entognatha	Collembola	Isotomidae		sp.1		X	P
Entognatha	Collembola	Paronellidae		sp.4	X	X	P
Entognatha	Collembola	Symphypleona		sp.2	X		E
Insecta	Blattodea			jovem	3		E
Insecta	Coleoptera	Scarabaeidae		jovem		X	P
Insecta	Dermaptera	Pygidicranidae		jovem	X		E
Insecta	Diptera	Cecidomyiidae	Cecidomyiinae	sp.	X		E
Insecta	Diptera	Muscidae		sp.	X		P
Insecta	Diptera	Psychodidae	*Psychodopygus*	Série *chagasi*	X		E
Insecta	Diptera	Psychodidae	*Sciopemyia*	*sordellii*		X	P
Insecta	Diptera	Tipulidae	Tipulinae	sp.	X		E/P
Insecta	Homoptera	Cixiidae		sp.6		X	P
Insecta	Hymenoptera	Formicidae	*Camponotus*	sp.1	X		P
Insecta	Hymenoptera	Formicidae	*Crematogaster*	sp.2	X		E
Insecta	Hymenoptera	Formicidae	*Solenopsis*	sp.1	X		E
Insecta	Isoptera	Termitidae	*Nasutitermes*	sp.	X	X	E/P
Insecta	Lepidoptera	Noctuoidea		sp.4	X		E
Insecta	Lepidoptera	Noctuoidea		sp.7	X		E
Insecta	Lepidoptera	Tineoidea		sp.5		X	P
Insecta	Neuroptera	Myrmeleontidae		jovem	2		E
Insecta	Orthoptera	Phalangopsidae	*Paraclodes*	sp.	2		E
Insecta	Orthoptera	Phalangopsidae	*Phalangopsis*	sp.	21	20	E/P
Gastropoda	Pulmonata	Bulimulidae	*Naesiotus*	sp.	X		E
Mammalia	Chiroptera	Emballonuridae	*Peropteryx*	sp.	4	6	E/P
Mammalia	Chiroptera	Phyllostomidae	*Carollia*	*perspicillata*	1		E
